# Conceptualising the social networks of vulnerable children and young people: a systematic review and narrative synthesis

**DOI:** 10.1007/s00127-020-01968-9

**Published:** 2020-11-02

**Authors:** Imogen Nevard, Chloe Green, Vicky Bell, Judith Gellatly, Helen Brooks, Penny Bee

**Affiliations:** 1 Division of Nursing, Midwifery and Social Work, Faculty of Biology, Medicine and Health, School of Health Sciences, University of Manchester, Manchester, UK; 2 Greater Manchester Mental Health NHS Foundation Trust, Manchester, UK; 3 Division of Psychology and Mental Health, Faculty of Biology, Medicine and Health, University of Manchester, Manchester, UK

**Keywords:** Social networks, Vulnerable children, Egocentric social network analysis, Wellbeing, Child health

## Abstract

**Purpose:**

The relationship between social networks and health and wellbeing is increasingly demonstrated in vulnerable adult populations. This relationship for vulnerable children and young people has not hitherto been systematically reviewed. This narrative synthesis aims to consolidate research to provide a foundational basis for future health-related social network research and interventions for children and young people.

**Methods:**

This mixed methods systematic review synthesises research investigating whole, egocentric social networks of 32 vulnerable child groups with a mean age below 18. There were no setting, language or date restrictions. The quality was assessed using the Mixed Methods Appraisal Tool. Of 6360 search results, 49 were included for narrative synthesis.

**Results:**

The majority of pertinent research originates from the USA; the most frequently investigated vulnerabilities were minority ethnic status, homelessness and the presence of special educational needs. Research aims and methodologies varied significantly between studies. Key findings included (i) vulnerable (excluding minority ethnic) children and young people have impoverished networks (ii) access to networks is a protective factor against negative outcomes (iii) social ties, primarily immediate family, provide access to personal resources and (iv) network ties are to a degree substitutable.

**Conclusions:**

Networks are associated with wellbeing and vulnerable children and young people commonly have impoverished networks, excluding cases where vulnerability classification relates to minority ethnic status. Network embeddedness is associated with positive outcomes, particularly for homeless children. Family are typically primary providers of support, but ties are substitutable when networks are restricted. Egocentric social network research is currently limited for vulnerable child populations. Further research could inform interventions that harness networks to improve health, wellbeing and functional outcomes for these child groups.

## Introduction

Vulnerable children and young people are at increased risk of poor health and quality of life-related outcomes, including mental health problems, physical illness and unhealthy lifestyle behaviours in childhood and beyond [27, 38, 96]. The Children’s Commissioner for England [77] definition of vulnerability, a problematic concept to characterise, is used here due to its unique specificity in delineating these vulnerable groups. The 2017 report defines vulnerable children as those at increased risk of adverse outcomes and identifies 32 child groups that meet this classification, falling under 9 domains: (i) Safeguarding concerns or in state care, (ii) health and/or disability (iii) economic circumstances (iv) family circumstances/characteristics (v) educational engagement (vi) involvement in offending or antisocial behaviour (vii) experience of abuse/exploitation (viii) missing/absent children (ix) minority populations.

Social approaches are common in health research with vulnerable children; frequently utilised concepts include socioeconomic inequality and access to social support. The World Health Organisation recognises the materiality of social factors to health, specifying how social contexts including family, geographic and economic patterns affect the health outcomes and behaviours of children and young people [19, 35]. The UN sustainable development goals demonstrate a clear commitment to strengthening health systems,social systems and informal networks are understood to play a key role in individuals interactions with treatment systems and execution of health behaviours [13, 57, 94], Pescosolido (1991). Currently progress toward these goals for children slow; 75% of child-related SDG indicators how insufficient progress or insufficient data [85]. The World Health Organization Mental Health Action Plan, extended this year to 2030, emphasises building social relationships as an indicator of child wellbeing [95].

This review utilises egocentric social networks as a conceptual framework for examining the social context of individuals. A social network is a personal community: the set of active and significant ties which are important to an individual’s everyday life [89]. An emergent body of research illustrates the role of networks in the aetiology and management of health conditions for adult populations [11, 81, 87]. Egocentric social network (ego-net) analysis collates data on the structure, function and composition of network ties of multiple individuals from the population of interest. Quantitative analyses often focus on modelling networks, where qualitative analyses such as inventory approaches focus on descriptive content regarding the quality and function of these ties; both are included in this mixed methods review. Network analysis is an underutilised method in child populations [70].

In this review we set out to determine the role and impact of social networks for vulnerable children and young people with particular respect to health, wellbeing and functioning outcomes. This study uses a broad concept of health as a quality of life informed phenomenon. This theoretically aligns with inclusive modern conceptions of health as more than an absence of disease, but as a relative state spanning physical, mental and social planes [76]. The review aimed to synthesise research using ego-net analysis to investigate the impact of social networks on the 32 identified vulnerable child groups. This study employs a mixed method narrative synthesis to identify common themes in descriptive information about vulnerable children’s networks, as well as associations between network variables and health- and wellbeing-related outcomes. This purpose of synthesising data from all identified vulnerable groups was two-fold (i) to provide baseline information that future research can compare or contrast demographic-specific findings to (ii) to provide comparable agglomerated data to base future hypotheses for groups where there is no current research. Flexible parameters were used to identify egocentric network methodologies, further explained in the discussion section of this manuscript. The review identifies gaps in this corpus of literature and potential entry points for health and wellbeing interventions.

### Background

Certain children and young people are at higher risk of adverse health- and wellbeing-related outcomes in both childhood and adulthood. Vulnerability is a frequently invisible, often intersecting and hard to quantify variable. Internationally, metrics for certain vulnerabilities have been created but estimates have not been effectively aggregated. However, the high proportion of children and young people exposed to adverse circumstance such as extreme poverty (19.5%), moderate to severe disability (5.1%) and residential care (2.7 m +) demonstrates the significant global scale of child vulnerability [29, 56, 84]. Additionally, other vulnerabilities may be underrepresented due to under identification resulting from social stigma, reluctance to access support or a structural lack of support available, as in the case of young carers of disabled and mentally ill relatives, children lacking formal identities or those caught up in armed conflicts [].

Social network analyses have been used to investigate the contagion of infectious disease, the spread of health-related behaviours and the impact of network density (how well-connected social actors are) on health outcomes [12, 14, 62, 68]. Causal mechanisms at work are yet undetermined,suggested influencing variables include social support, social influence, social engagement (identity and companionship), person-to-person contacts (exposure to infectious disease agents) and access to resources [7]. Some studies employ the notion of social capital, a contested term which describes the functional value of the structural systems involved in network formation and maintenance [1, 21, 64]. Of these varied definitions, this review aligns theoretically with Putnam’s definition *“features of social organization, such as trust, norms and networks that can improve the efficiency of society by facilitating coordinated actions” *[60]. Putnam’s interpretation of social capital allows for capital to be conceptualised on both the micro and macro level, as a feature of individual networks and of wider social systems. The role of capital as instrumental in the former is central to egocentric network level analysis. Proposed mechanisms by which social capital impacts health on an individual level include access to health information, informal provisions of care and support and the improved ability of cohesive groups to represent their needs and petition change [65].

Social network analysis is significant to vulnerable children and young people in two respects. Firstly, groups at higher risk of adverse outcomes are likely to have correspondingly higher levels of resource need. Networks are a major vehicle for resource access; vulnerable individuals may be highly reliant on network members for instrumental, emotional and informational and evaluative support [32]. Diversity of network members is an asset which allows individuals with vulnerabilities such as health conditions to access a range of different types of support [11, 89]. Substitutability between members suggests that networks can be a responsive system reactive to circumstance and need [87]. Reviews show that access to social capital predicts better mental and physical health [21]. Network embeddedness (the presence of a structure of close social ties able to facilitate resource flow around an individual) has been shown to have protective effect for vulnerable adults [16, 18, 39, 62].

Dependent on vulnerability, some individuals’ ability to access and maintain networks may be impaired, as demonstrated by studies linking vulnerability with low social connectedness or limited networks [4, 16, 17, 54, 93]. Several studies have demonstrated good outcomes from network diversification interventions for vulnerable adults such as those with mental health problems. A 2015 systematic review found that four out of five interventions aiming to increase network size for people with serious mental illness showed significant positive results [4]. Another recent systematic review also cautiously suggests that social participation interventions do appear to increase network size [90].

Application of this methodology to child populations is limited, with no systematic review consolidating existing research in this area [70]. It is can be inferred from promising health research with adults that social network methodologies could inform new avenues of positive health intervention for parallel child populations. Different internal factors such as cognitive development, communication and decision-making skills and external factors such as reliance on caregivers and socially undervalued rights and interests are likely to impact (i) how children and young people establish networks and (ii) how they participate in health research. For this reason, it is critical to examine children’s networks distinctly from caregivers or family units.

## Methods

This review was developed and carried out in line with the Preferred Reporting Items for Systematic Review (PRISMA) guidelines. The review protocol is available on the PROSPERO database, Reference Number: CRD4201801186. The process of narrative synthesis was informed by the ERSC Methods Programme Guidance on the Conduct of Narrative Synthesis in Systematic Reviews [59].

### Selection criteria

Empirical studies investigating ego-nets of 32 vulnerable child groups were included. Ego-net methods are those which collect information about network ties specific to one individual. The child groups identified by the Children’s Commissioner of England are shown in Table 1. Studies were included if the mean age was, or 75% of participants were, under 18. Studies were excluded if the age range spanned over 18 where no breakdown was provided. Both quantitative and qualitative studies were included if they used an egocentric approach. Studies were excluded if they only examined partial networks or one network member type. They were also excluded if networks were measured on a family-level. There were no restrictions on setting, language or date.

**Table 1:** List of vulnerable child groups

In care	In workless families	In ‘troubled families’^a^	Victims of modern slavery
Subject to Child Protection plans	Low-income	Parental substance misuse	Missing
Detained	Homeless	Parents with limited parenting capacity	Absent^b^
In need	Children assessed by social workers	Not in Education, Employment or Training	Minority Ethnic
Unaccompanied asylum seeking	Teenage parents	Excluded	Sex and gender minority
Care leavers	In non-intact families	Young offenders	Special educational needs/disability
Subject to Special Guardianship order	Undocumented	Gang members	Mental health difficulty
Adopted	Young carers	Trauma/abuse survivors	Physical health difficulty

^a^Children in families characterised by worklessness, children not being in school and family members being involved in crime and antisocial behaviour

^b^Children not where they are expected or required to be, such as missing persons

### Search strategy

CASSIA, British Nursing Index, CINAHL Plus, COCHRANE, Embase, ERIC, Medline, PsycINFO, Scopus Sociological Abstracts and Web of Science databases were searched for empirical research including peer-reviewed articles, conference presentations, books and dissertations. UK National Children’s Charity websites were also searched for grey literature but returned no relevant documents. Hand searches were performed of review papers identified in the initial search. A three stranded search parameter identified studies about (i) children, (ii) social networks and (iii) vulnerable child groups. Full search strategy can be found in Table 2.

**Table 2:** Search terms

Group A	Group B	Group C		
Adolescen*	“Personal communit*”	Vulnerable	Depriv*	“Health issue”
Child*	“Personal network*”	“At risk”	Homeless*	“Health concern”
Infant	“Personal ties”	At-risk	“Temporary accommodation”	“Health difficulty”
Min	“Social communit*”	Disadvantaged	“Insecure housing”	“Substance abuse”
Paediatric	“Social netwk*”	“Looked after”	“Unstable housing”	“Substance misuse”
Pediatric	“Social relationship*”	“In care”	“Sect. 17”	Alcohol
Teen*	“Social ties”	“Child protection plans”	“Social work assessment”	Drug*
“Young people”	“Relational netwk*”	Detention	“Teenage parent”	“Limited parenting capacity”
“Young person*”		Detained	“Teenage mother”	“Mental illness”
Youth		“Young offender”	“Teenage father”	“Mental ill health”
“Under 18”		“Youth offender”	Pregan*	NEET
“Under eighteen”		Custody	“Lone parent”	“Not in education, employment, training”
		“Secure training centre”	“Single parent”	Gang*
		“Secure children's home”	Divorc*	Trauma*
		Inpatient	“Non-intact family”	Slave*
		“In-patient”	“Non-intact families”	Missing
		Disabled	Undocumented	BME
		Disabilit*	Refugee	BAME
		“Social care”	“Young carer”	Black
		“In need”	Troubled	“Minority ethnic”
		Asylum	CRIME	LGBT
		“Care leaver”	Antisocial	Lesbian
		“Special guardianship order”	Exclusion	Gay
		Adopt*	Excluded	Bisexual
		Workless	“Domestic violence”	Trans*
		Unemploy*	Abuse	“Mentally ill parent”
		“Low income”	Neglect	“Parental mental illness”
		“Free school meals”	Maltreatment	
		Poverty	“Health problem”	

Searches were conducted in English only but all results were included.

### Screening

**Fig. 1:**
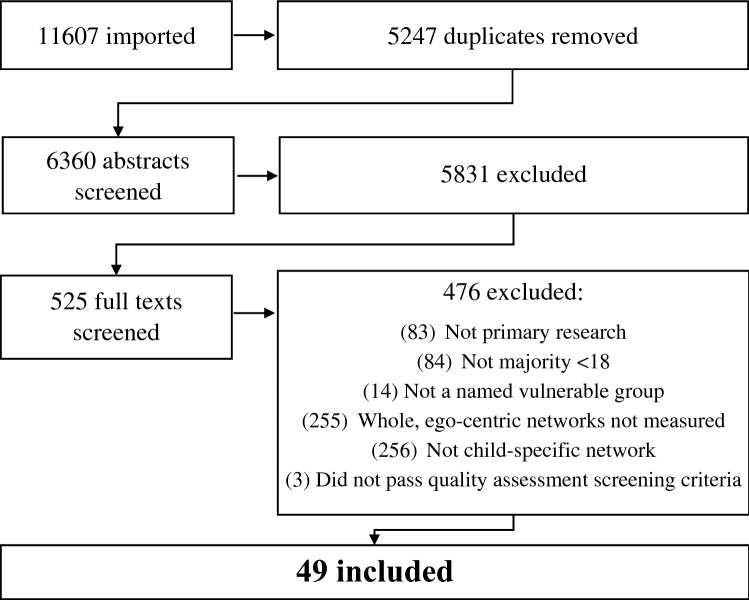
Systematic screening process

The initial search generated 6360 references. Titles and abstracts were screened independently by two reviewers. The most common reason for exclusion was misidentification due to the dual usage of the term social network to refer to both personal communities and digital social media. 5831 texts were excluded; full texts were acquired for 525 studies. Texts not in English were translated by colleagues from the first author’s research institution. Full texts were screened independently by two authors and disagreements resolved in the wider team. The MMAT [33] screening questions for inclusion in mixed methods systematic reviews were applied. 3 studies were excluded on this basis and 49 included in the final synthesis (Fig. 1).

### Data extraction

Data extraction was carried out by the first author using an extraction table formulated by the research team. Extracted data included sample size, setting, data collection and analysis methods and findings. Findings were translated into a common rubric of qualitative summaries as described in the Guidelines for Narrative Synthesis in Systematic Reviews [59].

### Narrative synthesis

Narrative synthesis was used to integrate data following the ERSC Methods Programme’s Guidelines for Narrative Synthesis in Systematic Reviews [59]. An initial thematic analysis provided a preliminary synthesis of data. Thematic analysis comprises line by line coding of this table of qualitative summaries and the development of descriptive themes based on this code. Units of meaning were identified and annotated; recurrent annotations were amalgamated and condensed into preliminary themes. Conceptual triangulation further explored relationships between key concepts, synthesising these using tabulation and conceptual mapping [24, 49]. This included diagrammatic exploration of the links between themes and revisiting the qualitative summaries and source manuscripts. Results are presented in narrative form. The qualitative data analysis software package NVivo was used to facilitate analysis.

## Results

Of the 6360 studies generated from the database search and reference checks, 49 met inclusion criteria.

### Quality assessment

Quality was assessed using the MMAT [33] tool. A rating out of 5 was awarded to each study based on the tool’s criteria for each respective methodology. Low quality studies were those with unclear aims that did not provide adequate descriptions of methodology. Studies with a score of five clearly outlined their aims, methods and analysis, explained the rationale for these choices and demonstrated the appropriateness of the techniques employed in generating their findings. Studies were only based on quality when they failed screening questions which would prevent successful data extraction. The rationale for this was the paucity of research targeting certain vulnerable groups; poor quality data could represent the totality of research.

Predominantly, samples were not representative of the population targeted. Aggregating samples across the 49 studies in this review is likely to minimise the impacts of this individual bias. Ethical considerations were not frequently reported in studies. Funding bodies were in some cases reported, but the risk of bias rarely considered. The exception to this was in dissertation studies where the potential impact of the role of the researcher on interview data was usually described.

### Overview of studies

A full overview of study characteristics is shown in Table 3. All collected data directly from children and young people except for two where parents were used as a proxy informant due to the child’s complex communication needs [61, 78]. An aggregated sample of 6364 vulnerable children and young people comprise the data set of this review. The weighted mean age across all 4645 participants in studies that reported breakdowns of participant age was 14.19 with a standard deviation 3.28. Controls not part of an identified vulnerable child group were excluded from this mean; age at entry point was used for longitudinal studies. 48% of the aggregated samples that provided this information were male and no studies allowed for gender reporting other than male/female.

**Table 3:** Descriptive information of included studies

	%
Country of origin	
Australia	6
Austria	2
Bangladesh	2
Belgium	2
Brazil	4
Canada	6
Kenya	2
Malawi	2
South Africa	2
Sweden	4
UK	2
USA	65
Vulnerability	
Minority ethnic	20
Homeless	12
Special educational need	12
Teen parent	10
In care	8
Abused/child protection	8
Non-intact families	6
Young offenders	4
Poverty	4
Mental health issue	4
Health condition	2
Asylum seekers	2
Methodology	
Quantitative	70
Qualitative	18
Mixed methods	12
Study design	
Cross-sectional	84
Longitudinal	16
Control group used	29
Quality rating	Total
5 = Excellent	6
4 = Very good	31
3 = Good	57
2 = Fair	4
1 = Substandard	0
0 = Poor	2

The most common vulnerability categories were ethnic minority status, homelessness and the presence of special educational need. Average network size across studies was 14.09 with a standard deviation of 12.09. Again, controls not part of an identified vulnerable group were excluded. An average was taken for longitudinal studies. The high standard deviation in network size reflects significant variation in ego-net measurement instrumentation.

### Findings on vulnerable children’s social networks

The full dataset of included studies including an abridged data extraction table can be found in Mendeley Data [https://dx.doi.org/10.17632/nwn9vcvcfd.3]. Narrative synthesis of these studies, grouped by themes, is presented below. These findings were frequent, although not ubiquitous across the data set. From a methodological perspective, ego-net methodology is not standardised. There is considerable variation in methods which was a barrier to synthesis. The themes describe different aspects of networks in terms of structure, utility and mechanism of effect. There were no consistent relationships with age or gender variables. Very few social network interventions have been trialled with under 18 s and no resultant significant health-related improvement evidenced. There is limited social network research on most vulnerable child groups, and methodology and research aim vary considerably between studies. Network findings aggregated by vulnerability are summarised in Table 4.

**Table 4:** Aggregated findings by vulnerability

Vulnerability	Studies	Total Sample^c^ (% male)	Weighted mean age (standard deviation)^d^	Weighted mean network members (standard deviation)^d^	Aggregated findings	Mean MMAT Score
Minority ethnic	[2, 3, 8, 9, 15, 44, 45, 53, 58, 75]	1372 (48.44)	15.33 (1.44)	23.17 (7.52)	Networks were significant sources of support. No findings regarding quality and composition of networks	3.4
Homeless	[22, 31, 37, 63, 67, 79, 80]	1695 (49.5)	16.61 (3.17)	13.01 (13.78)	Increased access to networks provided increased access to particularly instrumental support. Network embeddedness was typically associated with positive outcomes such as decreased arrest rate and risky behaviours	4.14
Special educational need	[23, 34, 41, 61, 78, 91, 92]	280 (60.72)	12.25 (1.88)	18.59 (46.00)	Children with special educational need have similar access to support. Differences in network size are inconsistently reported, but some studies report differences in network composition in terms of increased professionals and fewer peers	3.14
Teen parent	[26, 46, 73, 82]	427 (0)	16.31 (0.66)	6.13 (2.31)	All studies examined teen mothers. Family, primarily teen mother’s own mother are primary providers of support. Peers are also support providers. Social ties can also be a source of strain however	3.2
In care	[43, 74, 55, 86]	622 (38.8)	14.48 (2.85)	22.77 (7.28)	Children leaving care have smaller networks compared to non-care leavers; those with networks have better life satisfaction and lower depression scores	3.25
Abused/ child protection	[36, 50, 51]	706 (48.09)	10.9 (0.57)	15.59 (6.76)	Few consistent findings regarding network size or density	3.25
Non-intact family	[10, 20, 72, 97]	176 (58.43)	9.12 (1.85)	19.12 (0)	Networks are a key source of emotional/instrumental support. Support workers can be key providers of support; there is often more reliance on non-family adults	3.75
Young offenders	[52, 30]	100 (84.84)	15.93 (0.05)	8.66 (0)	Social support is protective against developing further vulnerability, in particular family support (particularly parents, especially mothers). Social workers are key support providers	2
Poverty	[5, 66]	316 (50)	14.36 (0.34)	12.86 (5.46)	Children subject to significant poverty have less access to support and fewer psychosocial needs fulfilled. Peer or school networks provide access to compensatory connections for these children. Network access is self-reinforcing	3
Mental illness	[42, 71]	504 (54.6)	14.78 (0)	4.79 (0)	Larger networks correlated with more support from services	3
Health	[48]	16 (50)	16.9 (0)	20.8 (0)	No difference in network quality or frequency of contact. Children with health condition had smaller networks with fewer non-related peers	3
Asylum	[47]	12 (100)	16.3 (0)	59	Professionals and ethnic community members are a significant source of support. Nonmigrant peers are not a source of support however	3

^c^Excluding non-vulnerable controls

^d^Where data are available

#### Impoverished networks

Studies commonly found that vulnerable children and young people had structurally impoverished networks, although metrics used were wide-ranging. Assorted measures demonstrated networks to be limited in terms of size, composition, interconnection and satisfaction. The ways in which networks were assessed to be limited included: a lack of social ties, a lack of specific social ties of one typology (such as peers or supportive adults), a lack of self-reported satisfaction or perceived interpersonal connection or tie closeness. All of these could represent a barrier to access of social support and limitation of social capital available through collaborative action between network members. This was found across the data set with the notable exception of studies where vulnerability related to ethnic minority status. Ethnic minority children and young people were the only group not to display impoverished networks. Vulnerabilities most commonly associated with limited networks were exposure to abuse or child protection issues or special educational need; children and young people had smaller networks when compared to controls who did not exhibit the explored vulnerability [23, 43, 48, 50, 61, 78].

In studies without a comparator group, vulnerable children and young people were frequently shown to have small or non-existent networks of zero to two people [22, 73]. Concerning composition, vulnerable children’s networks were often limited in peer contact, particularly for children with special educational needs. Some studies also observed vulnerable children and young people to have less dense networks, less frequent contact with network members and lower satisfaction with networks. Inter-study variation of methods limits specific structural conclusions about network restriction. However, the consistency in findings regarding restricted networks (excluding ethnic minority children and young people) across the dataset demonstrates that vulnerable children’s networks are frequently impoverished. Better standardisation of methods in future would allow for more accurate and specific structural comparisons.

#### Network embeddedness is correlated with positive wellbeing outcomes

Multiple studies correlated network embeddedness (the presence of a structure of close social ties able to facilitate resource flow around an individual) with positive wellbeing and functional outcomes. Network embeddedness was reported in varied ways depending on the data collection methods used, including factors such as: number of ties available, reported utility of ties, perceived closeness of ties available and diversity in network member type. Outcomes measured included: arrest rate, mental health, life satisfaction, access to social support, education, receipt of required services, psychosocial needs met, decreased risky behaviours and perception of risk and parenting ability. This was demonstrated across all groups except for children and young people with special educational needs or health conditions. Wellbeing-related outcomes were not investigated in these groups; instead studies contrasted network composition with non-disabled children and young people to generate structural conclusions.

Disparate research aims meant that few studies investigated equivalent outcomes, but in aggregate social network embeddedness improved wellbeing with regards to all outcomes measured except alcohol/substance use. Two studies correlated alcohol drinkers in network with alcohol use by the child; two found relationships to be protective or nil effect [22, 45, 58, 75]. Synthesis indicates that social ties are associated with positive wellbeing-related outcomes, potentially excluding contagion of harmful health-related behaviours within the network.

#### Social ties provide access to personal resources

Some studies investigated the process by which network embeddedness creates utility for vulnerable children. Most drew on notions of social support (or less frequently, social capital) to describe the instrumental, emotional and recreational resources flow between personal connections. Family, usually parents, were described as the primary source of (particularly instrumental) support [2, 15, 37, 40, 52, 55, 58, 67, 73]. Peers were also a significant source, although usually secondarily to family where the child had access to family ties [5, 15, 20, 40, 55, 63]. Professionals were occasionally, although not often, mentioned in networks,this was more common for children involved with governmental systems like asylum seekers or young offenders [47, 52]. Social networks are demonstrated to provide active, useful ties for vulnerable children and young people, facilitating assistive resource flow to realise positive wellbeing and functional outcomes. This cooperative action characterises successful social capital flows intra-network.

#### Substitutability of ties

The interpersonal mechanism of social support/resource flow is not clearly elucidated, although its utility is demonstrated. Flows of social support through social ties do not appear to be limited to specific network member typologies. The dataset demonstrated a degree of substitutability between different network members in relation to the support provided. The level and type of input from different network members was dynamic and responsive to individual circumstances. For example, where children and young people did not have access to traditional familial sources of social support (e.g. parents), it was often found that alternate ties could substitute for these close familial ties and provide compensatory support [5, 10, 20, 51, 55, 63].

The findings indicate that instrumental and emotional support were the most amenable to input from a wider set of network ties. This is particularly apparent in vulnerabilities that entail disrupted family networks such as non-intact families, homeless children and young people or those involved with child protection services. In these cases, other adults or peers were reported to provide instrumental and emotional support in lieu of close familial ties. Ego-net approaches can be valuable for identifying potentially substitutable ties. Developing interventions that aim to bolster such compensatory ties and offset negative outcomes associated with impoverished networks present an opportunity for the pragmatic integration of social network methodologies into health services for vulnerable children and young people.

## Discussion

In aggregate, this systematic review found associations between social network variables and wellbeing for vulnerable child groups. Vulnerable groups experience limited networks, with the notable exception of ethnic minority children. Most data relating to ethnic minority status sampled African American children and young people; it is unclear if this holds for other minority ethnic groups. Family are the primary source of intra-network social support, as is typical for non-vulnerable children [25]. However, vulnerability was often associated with disrupted access to typical network relationships. This review demonstrates that social ties can compensate for a lack of familial connections for these vulnerable children and young people. Networks are associated with a range of positive outcomes related to mental health, risky behaviour and academic outcomes.

There were no discernible gendered or age-related data trends. Few network interventions were identified in these searches for any vulnerable group; the two identified do not demonstrate effectiveness in altering networks and are based on inadequate data; one had a 98% attrition rate [36, 45]. Both interventions aimed to increase number of social ties through broader family or individual intervention programmes including social network counselling,neither study adequately described intervention methods. This indicates that network research for vulnerable children and young people has not reached a critical evidence base to underpin effective interventions targeting networks as a contributory factor to health wellbeing and functioning outcomes. There is limited social network research for most vulnerable child groups. None was found for many vulnerabilities including sex and gender minority status, exclusion or adoption. Ego-net methods were not standardised, and research aims, data collection and analysis methods varied considerably between studies.

### Speculative explanatory factors

This review finds that social networks and the health, wellbeing and functional outcomes for vulnerable children and young people are interrelated. Most research did not investigate directional effects or establish causal relationships. Potential mechanistic explanations for positive outcomes are manifold. Networks represent a vehicle through which individuals access social support; the connection of social actors through network ties generates social capital available to network members, although the interpersonal determinants of this (for example trust or norming) are not clearly specified. When present, family members are the primary source of this support which can be attributed to children’s increased dependence on others to meet their needs comparative to adults. Instrumental, emotional, informational and evaluative support access may directly mediate negative outcomes by preventing the need for engagement with risky behaviours.

Alternatively, the availability of social ties may impact the individual’s internal characteristics, such as self-esteem and confidence, in turn improving their coping ability and resilience. Conversely, vulnerable children and young people prone to negative outcomes could be less likely to engage with networks which offer a protective effect, limiting their potential to harness social capital through collaborative action.

The direction of causation between vulnerability and impoverished networks is equally unsubstantiated. Network creation could be impinged by the personal psychosocial capacity of the child or the resources the child has disposable toward the creation and maintenance of social ties. Alternatively, access to potential network connections could be limited due to contextual or locational restraints. These explanatory mechanisms are not sufficiently evidenced in the literature and at this stage can be only theorised. It is likely that no one hypothesis is explanatorily sufficient and all play a partial contributory role, varied subject to vulnerability type and individual characteristics.

### Methodological considerations

A broad understanding of social network research methodology was used in this research for a number of reasons. Firstly, there is not currently a standardised method used for egocentric network data collection. Although a diversity in research methods is not by default problematic, we found a clear lack in consistency in terms of quality of data collection and analysis. The divergence in methodology is a difficulty of the research area as a whole, and one of the key barriers to synthesis in this case. However, individual network analysis represents a promising avenue for health research in this area; a foundational review of even limited research provides an important cornerstone for this. Flexible parameters of network measurement were also used due to (i) the potential explanatory benefit of using both qualitative and quantitative data types and (ii) the general rarity of this methodology; this heterogenous collection of studies likely represents the full body of research available on this topic.

Methodological inconsistency across studies limit the specificity of findings. The disparate nature of aims, methods and outcomes prevent conclusive associations from being drawn between specific structural features of networks and vulnerability or outcome variables. Network metrics which assess networks as limited are rarely directly comparable. Studies measured varied factors including number of social ties, frequency of contact, closeness, overall satisfaction and supportive function of ties. Similarly, studies measured disparate outcomes. Although the most associated network embeddedness with positive outcomes, few used the same metric, or studied the same outcome.

Ego-net methods are considerably varied, and no standardised procedure is employed across the field. Methods that do not limit network members reported, that delineate between member typologies and that give a hierarchy of importance (often three tiered) are prototypical of exemplary egocentric social network analysis. Nunes et al. [52] is an example of best practice and methods reporting.

These limitations account for the scarcity of ego-net approaches in health interventions with vulnerable child groups. Standardisation of egocentric research methods, and the development of a methodologically consistent corpus of literature could underpin key developments in interventions and services targeting the health and quality of life of vulnerable child groups.

### Application and further research

Further research utilising ego-net approaches would be valuable to further evidence the relationship between network variables and health, wellbeing and functional outcomes for children and young people. There is a particular dearth of qualitative research useful for its explanatory power determining causal relationships. Research into the causal mechanism by which vulnerable children’s networks are impoverished is currently lacking.

The practical application of this evidence base is the development of social network interventions which can be used to improve outcomes, as for vulnerable adult populations [4, 90]. These may be particularly pertinent for homeless children and young people for whom the association between network-level variables and quality of life related outcomes is well evidenced.

The substitutability of social ties is particularly significant when developing interventions for groups with limited family networks. Interventions that target alternate network connections where support from family (the typical primary source) is limited, could prove effective for groups with disrupted networks, including children in care, with a mentally ill parent, or from non-intact families. There could be number of ways to integrate network factors into interventions for children and young people, either through bolstering internal psychological resources that help children and young people to access support around them, or external factors that assist in accessing support by facilitating connections to peers or professionals. Interventions could be particularly pertinent for children and young people with partially limited networks or without access to typical sources of instrumental or emotional support, who may be more reliant on sources outside of caregivers. These children and young people may be more reliant on compensatory connections where typical sources of social support are limited. In cases where children’s access to parental or peer group ties are limited, it is particularly relevant to consider the role that professionals could play as compensatory ties. Ego-net methodologies could be integrated at the assessment stage, to identify children most in need of network diversification interventions. Early stage monitoring of networks by involved professionals could prevent network shrinkage and avert network impoverishment over time.

### Limitations

PRISMA guidelines were employed for this review but some methodological limitations remain. Vulnerability is an amorphous term, making technical definition challenging. The Children’s Commissioner for England’s definition was used as the most clearly demarcated, due to its precise delineation of vulnerable groups used for estimation of national prevalence. This definition is superior due to its specificity, but the Anglo-centric bias in its formulation is a drawback. Vulnerability is a culturally varied concept, exacerbated where statutory-specific terms are employed. The Commissioner’s definition has been recently updated, demonstrating that vulnerability is likely to be an evolving concept. Furthermore, although texts in multiple languages were synthesised, relevant texts without a title or abstract available in English were not identified.

Classification by vulnerability was necessary to create a valid search strategy, but this overlooks that vulnerability is an intersectional phenomenon. Although studies were divided and synthesised based on the primary vulnerability investigated, other vulnerabilities were also present in samples. This prevented the establishment of more specific associations between vulnerability and network variables. This systematic review limited its scope to whole, egocentric social networks. A systematic review of partial or sociocentric (group-level) analyses was not pertinent to the research aim of investigating networks as an individual-level resource.

## Conclusions

Social networks impact the wellbeing of vulnerable children, as prior research shows for vulnerable adult populations. Vulnerable children and young people commonly have impoverished networks, excluding cases where vulnerability classification relates to minority ethnic status. Network embeddedness is associated with positive outcomes, particularly for homeless children and young people. Family are typically primary providers of support, but ties are substitutable when networks are restricted. Egocentric social network research is limited for vulnerable child populations. Further research could inform interventions that harness networks to improve health, wellbeing and functional outcomes for these child groups.

## Data Availability

The datasets analysed during the current study are available in Mendeley Data [https://dx.doi.org/10.17632/nwn9vcvcfd.3].
